# Mechanical Properties of 3D Printed vs. Subtractively Manufactured Composite Resins for Permanent Restorations: A Systematic Review

**DOI:** 10.3390/ma18050985

**Published:** 2025-02-24

**Authors:** Mauro Mandurino, Silvia Cortili, Luca Coccoluto, Katia Greco, Giuseppe Cantatore, Enrico Felice Gherlone, Alessandro Vichi, Gaetano Paolone

**Affiliations:** 1 Dental School, IRCCS San Raffaele Hospital, Vita-Salute University, 20132 Milan, Italy; mandurino200022@gmail.com (M.M.);; 2 Dental Academy, University of Portsmouth, Portsmouth PO1 2QG, UK

**Keywords:** 3D printing, mechanical properties, CAD/CAM, additive, subtractive, composite, resin-based

## Abstract

Objective: To conduct a systematic review on the mechanical properties of 3D printed resin-based composites when compared with those of subtractively manufactured resin-based composites. Materials and Methods: In vitro studies comparing the mechanical properties of additively and subtractively manufactured resin-based composites were sought. A systematic search, following the Preferred Reporting Items for Systematic Reviews and Meta-Analyses (PRISMA), was performed on four databases (PubMed, Embase, Web of Science, and Scopus) for articles published until 23 December 2024. The quality of the studies was assessed with the QUIN tool (risk-of-bias tool for assessing in vitro studies conducted in dentistry) and those assessed with a high risk of bias were excluded. Results: Of the 1058 screened articles, 13 were included in this review. A noticeable heterogeneity emerged in the methodologies employed, mainly regarding samples’ fabrication techniques, materials involved, and parameters analyzed. The most investigated mechanical property was fracture resistance, followed by microhardness, flexural strength, and wear behavior. Among the tested materials, the most used 3D printable resins were VarseoSmile Crown Plus (Bego) and Crowntec (Saremco Dental), whereas for the subtractive groups, the most investigated was Brilliant Crios (Coltène). Conclusions: The mechanical properties of 3D printed resins designed for permanent restorations are still lower than those of their subtractively manufactured counterparts. Moreover, in the long term, the degradation processes that inevitably occur might significantly increase their chances of failure.

## 1. Introduction

Aesthetic dental restorations are essential in modern dentistry, as they not only restore function and structural integrity but also improve patient satisfaction and overall well-being. This highlights the importance of biomimetic materials and advanced restorative techniques to achieve superior aesthetics and long-term clinical success [1,2,3]. The integration of dental 3D printing has revolutionized the fabrication of aesthetic restorations, enabling the precise customization of biomimetic prosthetics with enhanced accuracy, efficiency, and material optimization to meet both functional and aesthetic demands in modern restorative dentistry. Subtractive manufacturing (SM) has long been the dominant fabrication method in the computer-aided design (CAD) and computer-aided manufacturing (CAM) technology within the dental industry. This technology, which achieves the final product by milling a prefabricated block of material, has seen significant advancements in recent decades, particularly in dentistry. It is widely used for indirect dental restorations due to its good reproducibility, aesthetics, and reduced manufacturing time compared to other techniques [4,5]. SM can process a variety of restorative materials, such as ceramics, zirconia, resin-based composites, and hybrid materials. However, it does have limitations, such as the number of objects it can produce per machining operation and its inability to reproduce complex geometries [6].

On the other hand, the additive manufacturing (AM) process based on 3D printing technologies is emerging as a promising technique in the dental industry. Unlike SM, 3D printing technologies result from a buildup of consecutive layers. AM processes seem to have a significant number of benefits. The first and most immediate one is its ecological sustainability, provided by the reduced material waste [7]. It has lower manufacturing costs, and due to the possibility of printing numerous restorations simultaneously, it also results in a time-saving process.

Among the AM techniques used in the dental industry are stereolithography (SLA), digital light processing (DLP), and direct light projection using an LCD (liquid crystal display, also called mSLA: screen mask stereolithography apparatus) [8]. The American Section of the International Association for Testing Materials places DLP and SLA technologies within the same AM category because of their many similarities [9].

The oldest and most widely used method in dentistry is the stereolithography technique [9] because of the high precision and accurate building details it offers. Stereolithography is based on the buildup of consecutive layers of photo-sensitive liquid polymer, polymerized layer by layer using a focused ultraviolet light beam. The composition of the resins available on the market is homogeneous regarding photoinitiators and UV absorbers, enabling correct photopolymerization [10]. The properties of the printed layers, such as the thickness, direction, and angle of polymerization, are some of the factors influencing the mechanical properties of the final printed product [11].

Digital light processing (DLP) is an emerging 3D printing technology that uses a light source to cure photopolymer resin layer by layer two-dimensionally. Compared to SLA, which employs point polymerization by scanning laser, DLP cures a whole layer with a single flash of light, resulting in a time-saving process [12].

One significant advantage of both SLA and DLP is their compatibility with a wide range of resins available in the dental industry, which are consistent with photo-initiators and UV absorbers [13].

Currently, polymer-based materials account for most materials used in AM techniques [6]. The introduction of ceramic particles and nanoparticles to resin-based composites generated a new class of materials, the nanohybrid resin-based composites for 3D printing, which are claimed by manufacturers to be suitable for permanent restorations. The addition of inorganic fillers to the most commonly used filler-free resins modifies the overall mechanical properties of the final 3D printed product. Nevertheless, the printable materials need to maintain flowable consistency and structural stability during the printing process [14]. However, as 3D printing technology is gaining influence within the dental industry, uncertainties remain concerning the lack of evidence with respect to their mechanical properties, especially in terms of permanent restorations. Moreover, the lack of standardization in testing conditions often leads to conflicting results across studies. Given the rapid adoption of 3D printing in dentistry, investigating whether these materials are suitable for permanent restorations is both timely and necessary. Therefore, the aim of this systematic review is to evaluate the mechanical properties of printable resin-based composites designed for permanent restorations, focusing on studies that compare them to subtractively manufactured ones under consistent protocols and test conditions. The research questions identified by the authors of this review are: (i) how do the mechanical properties of printed RBCs compare to those of subtractively manufactured materials? (ii) what factors influence the mechanical performance of 3D printed resin-based composites? (iii) Are there significant limitations or advantages of using 3D printing technology for permanent dental restorations in terms of durability and clinical applicability? This systematic review is thus intended for dental researchers, clinicians, and professionals in restorative dentistry who seek to understand the comparative mechanical properties of 3D printed and subtractively manufactured resin-based composites, as well as for material scientists interested in advancements in dental fabrication technologies.

## 2. Materials and Methods

This review was conducted following the Preferred Reporting Items for Systematic Reviews and Meta-Analyses (PRISMA) Checklist (Support Information) [15]. The primary question was formulated according to the PICO strategy and served to develop the search strategy: (P)opulation: 3D printable resin-based composites for permanent restorations (I)ntervention: when their mechanical properties are tested (C)omparison: in respect to subtractively manufactured ones (O)utcome: how do they perform?

### 2.1. Information Sources

Two reviewers (M.M. and S.C.) conducted a search for English-language articles published until 23 December 2024 on the following databases: PubMed, Embase, Web of Science, and Scopus. A manual search was also conducted.

### 2.2. Search Strategy

Searches were performed by combining the following free text words and MeSH terms: ‘print’, ‘manufactur*’, ‘technology’, ‘printed-resin’, ‘SLA’, ‘DLP’, ‘printing, three dimensional’, ‘crown’, ‘restoration’, ‘inlay’, ‘onlay’, ‘overlay’, ‘veneer’, ‘flexural’, ‘fracture load’, ‘hardness’, ‘elastic modulus’, ‘mechanic*’, ‘stress’, ‘compressive strenght’, ‘fracture resistance’, ‘fracture toughness’, ‘fatigue’, ‘toughness’, ‘chip*’, ‘wear’, ‘mechanical properties’, ‘mechanical processes’, ‘peak stress’, ‘elastic strenght’, ‘microhardness’, ‘brittleness’, ‘flexibility’, ‘mechanical phenomena’, ‘stress, mechanical’, ‘mechanical tests’, ‘flexural strenght’, ‘elasticity’, ‘hardness tests’, ‘dental restoration wear’, ‘composite’, ‘resin based’, resin-based’, ‘resin’, ‘3D’, ‘three dimensional’, ‘addit*’, ‘stereolithography’, ‘interim’, ‘provisional’, ‘temporary’. All strategies were based on the search strategy developed for PubMed (Table 1) and revised appropriately for each database according to their controlled vocabulary and syntax rules. References of the included studies were also screened to identify relevant eligible studies.

### 2.3. Eligibility Criteria

Inclusion criteria:

1. In vitro studies investigating the mechanical properties of resin-based 3D printed and subtractively manufactured specimens.
2. Studies investigating resin-based materials designed for single-tooth permanent restorations.
3. Studies reporting numerical values of the investigated mechanical properties.
4. Studies written in English language.
5. Studies assessed with a low or medium risk of bias.

Exclusion criteria (The exclusion criteria were established to ensure the inclusion of only relevant, high-quality studies. Clinical trials, case reports, reviews, and animal studies were excluded as they introduce biological and procedural factors that could confound mechanical property assessments. Studies on temporary materials, dentures, surgical guides, or orthodontic attachments were omitted since the review focuses on resin-based composites for permanent restorations. Studies that did not report numerical values of mechanical properties were excluded to allow for quantitative comparisons. Additionally, studies assessed with a high risk of bias, as determined by the QUIN tool, were excluded to maintain the reliability and validity of the findings):

1. Clinical trials, case reports, reviews, or animal studies.
2. Studies investigating mechanical properties of temporary materials, dentures, casts, surgical guides, or orthodontic attachments.
3. Studies not reporting numerical values of the tested mechanical properties.
4. Studies assessed with a high risk of bias.

### 2.4. Selection Process

For the selection of studies, three authors (S.C., M.M., and L.C.) independently reviewed titles and abstracts of the retrieved records according to the inclusion criteria. Inter-rater reliability for study selection was assessed using Cohen’s kappa, which yielded a value of 0.78, indicating substantial agreement among the reviewers. The final inclusion of studies was based on full-text assessment and was performed with the consensus of all authors. In cases where discrepancies arose, a consensus meeting was held among the three reviewers. If disagreements persisted, a senior author (G.P.) acted as an arbitrator to make the final decision.

### 2.5. Data Items

A data extraction form was developed using a spreadsheet to record the variables extracted from the included papers to answer the research question. Retrieved variables included: permanent 3D printed resin-based material tested, milled resin-based material tested, printing method, printing layer orientation, printing layer thickness, specimen shape, specimen thickness, residual resin cleaning method, post-polymerization method, fatigue simulation, and mechanical properties tested.

### 2.6. Risk of Bias Assessment

Risk of bias was assessed using the QUIN tool (risk-of-bias tool for assessing in vitro studies conducted in dentistry) [16]. The quality assessment of the studies was based on a predefined set of bias domains (clearly stated aims/objectives; detailed explanation of sample size calculation; detailed explanation of sampling technique; details of comparison group; detailed explanation of methodology; operator details; randomization; method of measurement of outcome; outcome assessor details; blinding; statistical analysis; presentation of results). The final risk of bias assessment based on QUIN tool is reported in Table 2 and was obtained by categorizing all of the included papers as at ‘low’, ‘medium’, or ‘high’ risk of bias. Both reviewers (S.C. and M.M.) performed the assessment independently, and any disagreements or uncertainties were then discussed until an agreement was reached.

## 3. Results

### 3.1. Study Selection and Study Characteristics

**Figure 1:**
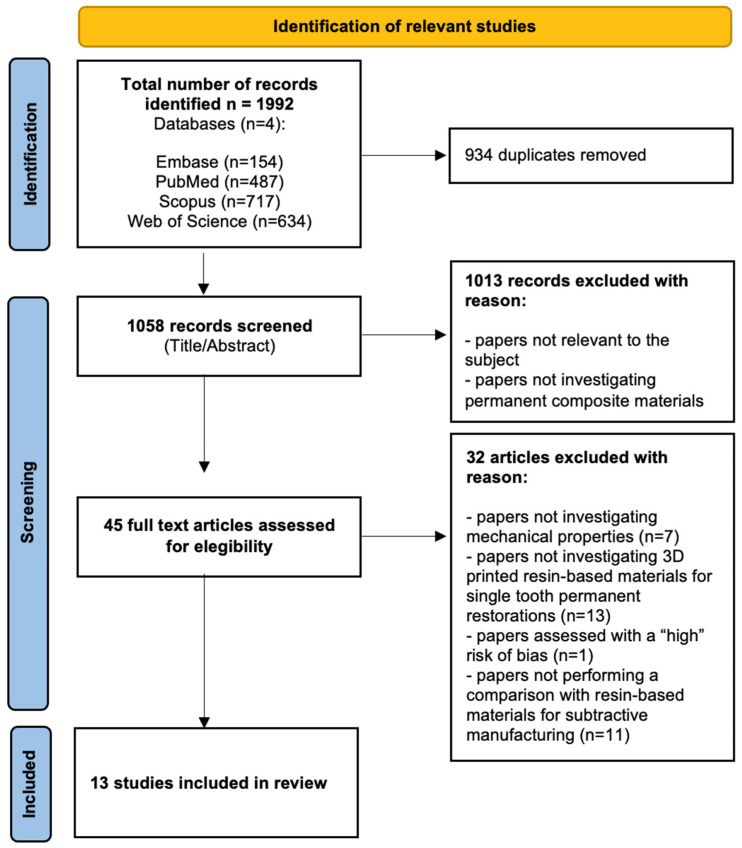
Identification of relevant studies.

The study selection process according to the PRISMA checklist is reported in Figure 1.

From the initial database search, 1992 articles were retrieved, and after duplicate removal, 1058 studies were screened based on their title and abstract. Out of these, 45 papers were selected for full-text evaluation. Finally, 13 studies [14,17,18,19,20,21,22,23,24,25,27,28,29] met the eligibility criteria and were included in this scoping review.

The variables of interest extracted from the included articles are reported in the above-mentioned predefined form (Table 3).

A noticeable heterogeneity emerged in the methodologies employed, mainly regarding samples’ fabrication techniques, materials involved, and parameters analyzed. Different mechanical properties of 3D printed permanent resin materials were examined, showing a proclivity toward fracture resistance (n = 6) [19,23,25,28,29], microhardness (n = 4) [14,17,22,27], wear behavior (n = 3) [20,21,29] and flexural strength (n = 3) [14,24,27]. Nevertheless, flexural modulus (n = 1) [14] was also investigated.

Among the tested materials, the most used 3D printable resins were VarseoSmile Crown Plus (n = 5; Bego, Bremen, Germany) and Crowntec (n = 5; Saremco Dental AG, Rebstein, Switzerland), followed by Permanent Crown (n = 3; Formlabs, Somerville, MA, USA), Tera Harz TC-80DP (n = 2; Graphy, Seoul, Republic of Korea), Iris Max (n = 1; DWS, Frankfurt, Germany), Els-3D Harz (n = 1; Saremco Dental AG, Rebstein, Switzerland), and C and B Permanent (n = 1; ODS, Incheon, Republic of Korea). Their composition is reported in Table 4. On the other hand, in terms of resin-based materials used for the comparison with subtractive manufacturing, the most investigated was Brilliant Crios (n = 8; Coltene, Altstätten, Svizzera), followed by Grandio Blocs (n = 4; Voco, Cuxhaven, Germany), Cerasmart (n = 4; GC Corporation, Tokyo, Japan), Lava Ultimate (n = 1; 3M Espe, St. Paul, USA), Mazic Duro (n = 1; Vericom, Chuncheon, Republic of Korea), and Cerasmart (n = 1; GC Corporation, Tokyo, Japan).

### 3.2. Mechanical Properties Data

Although the different experimental designs do not allow a reliable comparison, values from the included studies have been added to Table 5, Table 6 and Table 7 to provide a detailed overview of the mechanical properties of 3D printed and subtractively manufactured resin-based composites. Table 5 presents fracture resistance values across different materials and thicknesses, highlighting variations in load-bearing capacity and the impact of specimen thickness on performance. Table 6 summarizes microhardness measurements, demonstrating consistent trends of lower hardness in 3D printed resins compared to their milled counterparts. Table 7 reports flexural strength values, further confirming the generally superior mechanical performance of subtractively manufactured materials. These tables offer a structured and quantitative representation of the findings, enhancing the comparability of results and supporting the observed trends in mechanical behavior.

#### 3.2.1. Results of Studies Analyzing Fracture Resistance

Among the included studies analyzing the fracture resistance of additively and subtractively manufactured resin-based materials, opposite results were reported when the influence of specimens’ thickness was considered (Table 5). Most studies testing various thicknesses have consistently reported a positive correlation between thickness and fracture resistance [18,25,28]. Nevertheless, Park et al. [23] found that, while this correlation was confirmed for milled specimens, an increase in thickness for 3D printed specimens was associated with a decrease in the load required to cause fracture.

Furthermore, conflicting results were found when evaluating the material effect on load-to-fracture values. Corbani et al. [18] reported that the additively manufactured group showed higher fracture resistance values compared to the milled group, regardless of the materials’ thicknesses: 0.5 mm (*p* < 0.001), 1 mm (*p* < 0.001), and 1.5 mm (*p* < 0.001). Moreover, at a thickness of 0.5 mm, the lowest mean difference between the two groups was recorded.

In contrast to these findings, Donmez et al. [19] and Zimmermann et al. [28] did not find any statistically significant differences in terms of fracture resistance between the 3D printed group and the subtractively manufactured group. However, even though the analyzed thicknesses were the same, the materials tested and the study designs differed.

Turksayar et al. [29], who tested two different resin-based composites for AM, reported that one (VarseoSmile Crown Plus; Bego) had similar values to those of the milled one (Brilliant Crios; Coltene), whereas the other (Crowntec; Saremco Dental AG) showed significantly lower values. However, no information was reported about specimens’ thickness.

Accordingly, Suksuphan et al. [25] reported that all of the 3D printed crowns fractured at a certain compressive force (1480 ± 226 N; 1629 ± 118 N; 1747 ± 108 N, respectively, at 0.8, 1, and 1.5 mm thicknesses), while the milled resin-based composite could withstand the maximum load (<2000 N).

Finally, Park et al. [23] reported that 3D printed materials demonstrated higher fracture resistance at thicknesses of 0.5 mm (3770 ± 613 N) and 1 mm (2677 ± 398 N), whereas the milled counterparts exhibited greater strength at a thickness of 1.5 mm (3218 ± 228 N).

#### 3.2.2. Results of Studies Analyzing Microhardness

All included papers analyzing the microhardness (Table 6) of resin-based composites agree on the fact that 3D printed resins presented significantly lower values compared to milled ones. For the AM specimens, the Vickers microhardness values were similar in all studies (~30 VHN), whereas for the subtractively manufactured resin-based composites, these values ranged from 75.4 ± 2.2 VHN (Brilliant Crios; Coltène) [14] to 203.9 ± 3.6 VHN (Grandio Blocs; Voco) [22]. Grzebieluch et al. [14] compared the microhardness of the same 3D printable resin-based composites (VarseoSmile Crown Plus; Bego) produced with two different printing angles (vertical to the platform and 45 ∞) and no statistically significant difference between these two groups was found. Furthermore, all papers that performed hydrothermal aging [17,22,27] align with the fact that no significant effect was observed on the microhardness of tested resin-based composite specimens.

#### 3.2.3. Results of Studies Analyzing Flexural Strength

Two [14,27] of the three studies that analyzed flexural strength (Table 7) used the 3-point bending test, whereas the third study [24] performed the biaxial bending test. Nevertheless, independently from the study design, all of them reported higher flexural strength values for the milled groups compared to the 3Ds. Moreover, from the above-mentioned comparison between two different printing angles, Grzebieluch et al. [14] found a significantly higher flexural strength for the 45 ∞ group. Nonetheless, these values were still inferior compared to those of the milled resin-based composite. In the same study, the flexural modulus was also calculated; however, no statistically significant difference was found between the two printing angles.

Grzbieluch et al. [14] reported values of initial flexural strength ranging from 120 MPa to 140 MPa based on printing orientation. Prause et al. [24], on the other hand, reported an initial value of 80 MPa, and Yilmaz et al. [27] reported values ranging from 90 MPa to 105 MPa.

Prause et al. [24] included a fatigue simulation using the staircase method and found that the 3D group exhibited a significantly higher strength degradation (55.2%) compared to the milled resin-based composite group (40.5%). Furthermore, Yilmaz et al. [27], who performed hydrothermal aging, reported a significant decrease for the subtractively manufactured group (from 211 ± 17 MPa to 172 ± 19 MPa) and for one of the two 3D printed resin-based composites tested (Tera Harz TC-80DP; Graphy; from 108 ± 16 MPa to 85 ± 4 Mpa). Nevertheless, no significant difference was found between the flexural strengths of the two 3D printed resins.

### 3.3. Results of Studies Analyzing Wear Behavior

A conflict between the results of the included studies analyzing wear behavior was found. Guven et al. [21] reported, in fact, that the milled resin-based composite (Brilliant Crios; Coltene) presented a higher volume loss in the worn area compared to the 3D printed one (Crowntec; Saremco Dental AG). On the other hand, Turksayar et al. [29], who analyzed the same materials as well as another additively manufactured resin-based composite resin (VarseoSmile Crown Plus), found that the two 3D printed groups were more prone to abrasion, both in terms of volume loss and maximum wear depth. Finally, Guntekin et al. [20] observed that the 3D printed group (Permanent Crown; Formlabs) exhibited comparable volumetric wear values to one of the milled groups (Grandio Blocs; Voco), while showing higher wear compared to the other milled group (Cerasmart; GC).

### 3.4. Sensitivity Analysis

A sensitivity analysis (Table 8) was performed to assess the influence of medium risk studies on the retrieved results. The sensitivity analysis was performed using a statistical software (STATA 14.1; StataCorp LLC, Lakeway Drive, TX, USA), which allowed for systematic exclusion of medium risk studies and recalculation of mean values for key mechanical properties to assess their impact on the final conclusions. The differences in fracture resistance, flexural strength, microhardness, and wear behavior are minimal, indicating that the medium risk studies do not significantly bias the final conclusions.

### 3.5. Correlation Analysis

To assess the impact of filler content and type on the mechanical properties of 3D printed and subtractively manufactured resin-based composites, a correlation analysis (Table 9) was conducted using (STATA 14.1; StataCorp LLC, Lakeway Drive, TX, USA). The analysis evaluated the relationships between filler content (%) and key mechanical properties, including fracture resistance (N) and flexural strength (MPa).

The correlation analysis revealed a strong positive correlation (r = 0.88) between filler content and fracture resistance, suggesting that materials with higher filler concentrations exhibit greater resistance to fracture. This trend aligns with prior findings indicating that a higher proportion of inorganic fillers enhances mechanical stability by increasing the material’s elastic modulus and reducing polymerization shrinkage.

Similarly, a moderate positive correlation (r = 0.67) was observed between filler content and flexural strength, indicating that higher filler content generally contributes to improved resistance to bending forces.

Furthermore, a correlation analysis was also conducted to assess the relationship between curing mechanisms and key mechanical properties (fracture resistance and flexural strength) in 3D printed and subtractively manufactured resin-based composites. The analysis utilized categorical encoding for different curing methods and examined their correlation with the mechanical properties. The results indicate a moderate positive correlation (r = 0.39) between curing mechanism type and fracture resistance, suggesting that heat-assisted and xenon lamp curing methods contribute to enhanced resistance against fracture. This trend aligns with previous findings that thermal curing improves polymer cross-linking, resulting in a more robust material structure capable of withstanding masticatory forces.

Conversely, a moderate negative correlation (r = −0.53) was observed between curing mechanism type and flexural strength, indicating that certain curing methods (e.g., UV or purely light-based curing) may lead to reduced flexural performance.

## 4. Discussion

The evolution of aesthetic dental restorations has progressed from traditional direct resin-based composite techniques, which rely on manual layering and sculpting, to the advent of CAD/CAM and, more recently, 3D printing technologies, that offer enhanced precision, material efficiency, and reproducibility in fabricating highly aesthetic and functionally durable restorations [11,30]. The introduction of this new technology as an alternative to SM has become increasingly popular. This is mainly due to its lower cost and the possibility of saving energy and materials, therefore reducing the carbon footprint [12]. AM is similar to subtractive processes in terms of impression techniques and fabrication steps. However, there is much more waste deriving from milling procedures since this technique makes use of different burs to cut prefabricated blocks or disks, which are non-reusable and must be, therefore, disposed of.

Thanks to the advantages brought by 3D printing, the dental industry is constantly releasing new products, seeking to improve the mechanical and aesthetic properties of these new materials. The first printable resins marketed were designed for interim restorations only, mainly due to the absence or low presence of filler [31]. Nowadays several manufacturers have promoted new resins, which are claimed to be appropriate for permanent restorations, but scarce information is present in the literature about their mechanical properties, especially in comparison to their milled counterparts.

In our review, relevant, and comparable studies that specifically address the mechanical properties of resin-based 3D printed and subtractively manufactured materials for permanent dental restorations were included. Clinical trials, case reports, reviews, or animal studies were excluded, as these types of studies may not provide standardized, controlled mechanical testing of the materials. Furthermore, to provide meaningful comparisons, the review requires measurable, quantitative data (e.g., flexural strength, fracture resistance) rather than qualitative descriptions or subjective assessments.

One of the main properties that categorize a material as suitable for permanent restorations is fracture resistance. These materials should in fact be able to withstand masticatory forces, which have been reported to reach 424–583 N in the premolar region [32] and up to 900 N in the molar area [33]. These values could also increase for patients with implant-supported restorations due to the lack of proprioception, as well as for patients with bruxism [34].

Different study designs were found among the included papers analyzing fracture resistance. Nevertheless, all of them agreed that with a minimum thickness of 1.5, all tested resin-based composites exceeded the above-mentioned occlusal load values. Material thickness is, in fact, one of the main factors influencing fracture behavior, and its impact has been investigated by some of the articles included in this review. Most of them [18,25,28] reported that a positive correlation could be found between the material’s thickness and fracture resistance.

Nevertheless, in some clinical situations, such as occlusal veneers or minimally invasive crowns, it might be necessary to produce extremely thin restorations, even though thicknesses of less than 1.5 mm are not recommended by the manufacturers. When 0.5–1 mm thick specimens were tested, various results were found among different studies and materials. Corbani et al. [18] reported that 3D printed specimens (Iris Max; DWS) outperformed those produced by subtractive manufacturing (Brilliant Crios; Coltène). The latter, in fact, exhibited a relatively low resistance to fracture (519,3 N) at the thickness of 0.5 mm, which increased for thicker specimens (1 mm = 923,1 N; 1.5 mm = 1284.7 N). The 3D group, instead, proved more suitable to withstand occlusal loading; even with a lower thickness (0.5 mm), it exhibited a high range of resistance to fracture (1345 N). The authors attributed these findings to the tools and processes employed by the subtractive method, which may result in a weakening of the material, thereby increasing the risk of fracture. The process of trimming resin-based composite blocks or disks may in fact lead to the creation of flaws on the restoration’s surface, which eventually result in crack formation. Moreover, the incidence of these deformities seems to depend on the direction, depth, speed, and wear of the burrs, as well as on the cutting configuration, namely down or up milling [35].

Park et al. [23] reported results similar to those of Corbani et al. [18], showing significantly higher fracture resistance values for 3D printed specimens compared to milled ones at low thicknesses. However, in contrast to Corbani et al. [18], Park et al. [23] observed that the fracture resistance of AM specimens decreased with increasing specimen thickness. The authors attributed these findings to the large molecular weight of the composed oligomer and its flexibility, which they explained as being due to the low cross-linking density, accompanied by high deformation characteristics. In this context, 3D printed resin-based composites might be preferred for minimally invasive restorations that require low thicknesses.

On the other hand, Suksuphan et al. [25] reported that milled resin-based composites presented higher fracture resistance compared to the 3D printed group, and motivated their results, indicating that the cause may lie on the materials’ composition. The additively manufactured resin-based composites, in fact, present lower filler content in respect to the milled ones, which may be the cause of a lower flexural strength and fracture resistance. The different designs and materials tested do not allow a direct comparison between the two studies. However, all resin-based materials tested, both additively and subtractively manufactured, at all thicknesses (including 0.5 mm), presented fracture loads superior to the above-mentioned occlusal load values.

Donmez et al. [19], on the other hand, in line with Turksayar et al. [29] and Zimmermann et al. [28], found that independently of their different study designs, 3D printed resin-based composites showed similar or slightly worse fracture behavior than those produced via subtractive manufacturing. In addition, those studies that performed thermomechanical aging [28,29] obtained inferior fracture load values compared to those that did not, and these values were lower than the above mentioned mastication forces, indicating that in the long term, the risk of fracture might increase, especially for thin restorations.

It is important to emphasize that to properly evaluate the results, the entire study setup must be considered, making this type of study extremely complex to compare. The E-modulus of the die, for example, has shown to play an important role in determining flexural fracture [36]. Moreover, the fracture load of monolithic ceramic crowns has been shown to increase linearly with the increase of the elastic modulus of the supporting material [37]. This might explain the opposite results obtained by Corbani et al. [18], Park et al. [23] and Suksuphan et al. [25] as the first one used an SLA-printed resin with a modulus of elasticity of 3 GPa to fabricate the abutment die, whereas the second one used metal dies, and the last one a harder resin (9 GPa of E-modulus). The latter, however, is the most suitable to simulate clinical conditions [38] since the E-modulus of natural dentin has been reported to be between 7 and 13 GPa [39].

Another important mechanical property is flexural strength. The minimum value for polymer-based restorative materials to be suitable for “restorations involving occlusal surfaces and whose use requires the energy to be applied extra-orally” is reported by the ISO standard 4049:2019 to be 100 MPa (type 1, class 2 materials). This ISO standard was taken into consideration in the present study since a more specific one for 3D printed materials is not yet available. Moreover, the same threshold (100 MPa) is also required for “monolithic ceramic for single-unit anterior or posterior prostheses adhesively cemented”, as reported by the ISO standard 6872:2015 and therefore, applies to both additively and subtractively manufactured specimens considered in the present study.

All of the included articles [14,24,27] analyzing flexural strength reported that superior flexural strength was found for milled specimens compared to additively manufactured; nevertheless, values satisfied critical mechanical ISO benchmarks. This finding was mainly ascribed to the lower filler load (30–50 wt %) of 3D printed resin-based composites which has been shown to be linearly correlated with lower flexural strength [40]. This low percentage of inorganic fillers is motivated by the fact that a higher filler ratio would increase the risk of void incorporation due to the limited or impaired resin’s flow during the layering process, possibly lowering the mechanical properties [41]. It is worth noting that Prause et al. [24] reported that with this concentration, no voids were observed under the SEM and that fracture origins were identified in areas of non-homogenous microstructure, which the authors reported to arise from an uneven mixing of the resin-based composite’s components.

While most studies included in this review followed standardized testing protocols, such as the three-point bending test outlined in ISO 4049, discrepancies in loading conditions and aging procedures may have contributed to differences in mechanical outcomes. For example, Prause et al. [24] employed a biaxial bending test, which is known to produce higher flexural strength values due to its ability to distribute stress more evenly across the specimen and reduce edge effects that could initiate failure. In contrast, Grzebieluch et al. [14] and Yilmaz et al. [27] utilized the three-point bending test, which subjects the specimen to peak tensile stress at the bottom surface, making it more sensitive to surface flaws and stress concentration effects. This difference in testing methodologies likely accounts for the higher flexural strength values reported by Prause et al. [24] compared to those obtained in studies using uniaxial bending tests.

Additionally, environmental conditioning plays a significant role in material performance. Studies incorporating hydrothermal aging, such as those by Yilmaz et al. [27] and Turksayar et al. [26], reported a noticeable decrease in flexural strength and microhardness, highlighting the susceptibility of 3D printed resins to water-induced degradation. The absorption of water into the polymer matrix can lead to plasticization and hydrolysis, resulting in reduced mechanical integrity over time. Conversely, studies that omitted aging procedures, such as Grzebieluch et al. [14], generally reported higher initial flexural strength values, further emphasizing the impact of environmental exposure on long-term material performance. The lack of uniformity in aging procedures among studies underscores the need for standardized preconditioning protocols to better simulate intraoral conditions and improve the comparability of results. Future research should aim to integrate consistent aging methods and standardized loading conditions to enhance the clinical relevance of flexural strength assessments in 3D printed dental materials.

Another drawback resulting from the large amount of resin phase contained in 3D printed resin-based composites is the high susceptibility to strength reduction, reported by Prause et al. [24] to be 5% higher compared to the subtractively manufactured group, mainly due to water sorption, creep deformation, and hydrolysis [42]. It was, in fact, reported that hydrothermal aging may induce water sorption, which degrades polymeric chains and reduces their mechanical properties, due to the plasticizing action of water [43]. Yilmaz et al. [27] supported these results, noting that subtractively manufactured resin-based composites may have appeared to be less prone to hydrolytic degradation due to their densely cross-linked and homogeneous structure, resulting from a higher filler percentage and the higher degree of conversion obtained by the polymerization performed under controlled light and pressure conditions. Moreover, from the same study it was reported that the previously mentioned threshold of 100 MPa was obtained by most of the SM groups but not by the AM materials when subjected to aging conditions. When considering non-aged conditions, only one of the two additively manufactured resins reached values higher than 100 MPa, likely depending on the differences in chemical composition [27].

Grzebieluch et al. [14], in contrast to these findings, reported values of flexural strength for additively and subtractively manufactured specimens higher than this threshold. In the same research paper, the printing orientation was also examined, and an association with flexural strength was found. When the printing angle was set at 45°, in fact, higher flexural strength was recorded in comparison to the 90° printing angle. This finding is in line with a previous study [44], in which it was reported that 3D printed restorations at 30°, 45°, and 60° showed higher flexural strength compared to those manufactured at 0° and 90°. However, regarding the printing orientation, differing results are reported in the literature in terms of flexural strength, mainly related to the materials tested, the adhesion surface dimension, and the polymerization rate [45]. Moreover, higher flexural strength results from horizontal orientation compared to vertical orientation [45,46]. In the second case, in fact, the load direction becomes parallel to layer interfaces, which is associated with reduced flexural strength [45] due to the weaker adhesion obtained between layers compared to that present within the same layer [47].

It is worth noting that the study design played an important role in determining the final values of flexural strength. First of all, the ISO standard 4049 requires the use of the 3-point-bending test, which was only used by Grzebieluch et al. [14] and Yilmaz et al. [27]. Moreover, it also requires a specimen thickness of 2 mm whereas in these two studies a thickness of 1.5 mm was considered. This must be kept in mind when their results are compared with the previously mentioned threshold, since the specimen’s thickness is expected to alter flexural strength values [45]. Furthermore, when taking into consideration the results of Prause et al. [24], it must be underlined that they used the biaxial bending test. The latter was reported to produce higher values of flexural strength compared to the 3-point-bending test, owing to a smaller stress zone. This reduces the stresses generated in the defective volume and therefore lowers the influence of cracks and flaws present at the specimen edges [48,49]. Furthermore, also the different procedures of specimen preparation may have had an influence on the outcome. For example, the higher values reported by Grzbieluch et al. [14] may be due to the absence of a storage step in distilled water, as performed by Yilmaz et al. [27] and Prause et al. [24] The latter performed the biaxial bending test. Despite this, they reported the lowest values, but it should be noted that they stored the specimens for 14 days, whereas Yilmaz et al. [27] did so for 24 h. The above-mentioned processes of hydrolytic degradation may have played a role in the differences observed between these three studies. For these reasons, more studies are needed in order to properly evaluate the flexural strength of 3D printed materials. Moreover, a standardization of methods is strongly advisable.

Another mechanical property investigated by the studies included in this review is microhardness, which is critical for permanent restorations because it relates to the durability and clinical behavior of the material. An agreement was found in literature on the fact that SM allows for a greater surface hardness compared to 3D printing [14,17,22,27]. The main reasons were once again the lower content of inorganic filler and the different polymerization process, with the latter leading to inferior degrees of conversion of the organic matrix and therefore to inferior microhardness [50]. Even though some strategies to increase microhardness have been proposed in the literature, such as an increase of post-curing time [51] or a different printing orientation [14], currently, none of them allow 3D printed resins to reach the values of manufactured resin-based composites. Consequently, since surface hardness and wear behavior are related, a greater volume loss would be expected for additively manufactured resin-based composite materials. Surface finish inconsistencies can significantly influence wear behavior, particularly in 3D printed materials where post-processing techniques vary. Differences in polishing, curing, and post-printing treatments may affect surface roughness and, consequently, the wear rates reported in different studies. While some included studies followed standardized finishing protocols, others lacked detailed descriptions of these procedures, potentially contributing to variability in the results. Unfortunately, once again conflicting results were reported by the only two articles that performed this comparison. Guven et al. [21] reported that a greater volume loss was observed for the subtractively manufactured group, whereas Turksayar et al. [29] stated that 3D printed specimens had higher volume loss and maximum wear depth compared to the milled ones. This difference in results was ascribed by Guven et al. [21] to the different designs of tested specimens and to the different scanning devices used, reporting that the intraoral scanner they used was more precise than the laser scanner used by Turksayar et al. [29] However, the inferior wear resistance of AM materials is an expected consequence of their lower microhardness. As confirmed by Guntekin et al. [20], the reduced filler load and larger filler dimensions increase the intermolecular distance, thereby enhancing their susceptibility to abrasion and resulting in greater volume loss [52].

Only VarseoSmile Crown Plus (Bego) showed a significant number of investigations; therefore, a profile of this 3D printable resin can be drawn. This material is claimed to be indicated for single permanent crowns, and its flexural strength and fracture resistance were shown to be sufficient immediately after printing [14,24,25,29]. Conversely, it emerged that in the long term its mechanical properties drop, mainly due to hydrolytic degradation of the resin matrix [17,24,29]. After prolonged immersion in distilled water or thermocycling, in fact, significantly lower flexural strength, fracture resistance, and microhardness were reported [17,24,29], the latter resulting in a higher susceptibility to surface wear [29]. Permanent materials for 3D printing are clearly still in their infancy, and in vitro and in vivo studies are strictly necessary to clarify the clinical indications of this material as well as those of its competitors. The findings of this systematic review highlight the ongoing debate regarding the mechanical reliability of 3D printed resin-based composite resins for permanent restorations compared to their subtractively manufactured counterparts. While additive manufacturing (AM) has demonstrated advantages such as cost efficiency, material conservation, and increased customization, its clinical applicability remains limited by concerns over mechanical performance. Across the reviewed studies, 3D printed resin-based composites consistently exhibited lower fracture resistance, microhardness, and flexural strength than milled materials, which raises concerns about their long-term durability in the oral environment, especially under high occlusal forces. These mechanical deficiencies are largely attributed to differences in polymerization processes, filler content, and the intrinsic layering structure of AM materials, which may introduce inherent weaknesses in the final restoration.

One of the major discrepancies observed in the literature pertains to fracture resistance at varying thicknesses. While some studies indicated that 3D printed materials could perform comparably to subtractively manufactured resin-based composites at low thicknesses, others demonstrated a decline in mechanical strength with increased material thickness. This suggests that AM resins may have a specific thickness range in which they perform optimally, but further research is needed to establish these parameters. Similarly, hydrothermal aging and long-term degradation were found to negatively impact AM materials more significantly than milled resin-based composites, suggesting that while they may be suitable for short- to mid-term applications, their long-term reliability in permanent restorations remains uncertain.

From a clinical perspective, these findings underscore the need for cautious integration of 3D printed resins into routine dental practice, particularly for stress-bearing restorations. While they may be suitable for certain indications, such as single-unit restorations in low-load areas or temporary restorations, their widespread use in high-load-bearing regions (e.g., molars) may require further material advancements. Additionally, given the variability in reported results, standardized testing protocols are crucial for ensuring consistency and comparability across future research. The implementation of uniform mechanical testing methods, longer aging simulations, and clinical trials will be essential in determining whether AM resins can truly replace milled resin-based composites in permanent restorations.

A key limitation in the evaluation of wear behavior in 3D printed resin-based composites is the potential influence of surface roughness, which may vary due to differences in post-processing techniques. While wear behavior was assessed in the included studies using methodologies such as volumetric wear analysis and profilometry, the impact of surface roughness was not consistently reported or controlled. Given that 3D printed materials often exhibit higher initial surface roughness compared to their subtractively manufactured counterparts, variations in post-processing—including polishing, ultrasonic cleaning, and curing protocols—may contribute to discrepancies in wear performance across studies. Some studies employed standardized finishing protocols, whereas others provided limited details on surface preparation, making direct comparisons challenging. Since surface finish inconsistencies can significantly affect wear resistance, future studies should incorporate uniform post-processing methods and explicitly report surface roughness parameters to improve the reliability and comparability of wear data in additively manufactured dental materials.

In conclusion, while 3D printing presents a promising direction in restorative dentistry, its current mechanical limitations suggest that further improvements in material formulation, polymerization techniques, and testing methodologies are necessary before these materials can be widely adopted for permanent applications. The results of this review indicate that standardization of testing procedures and long-term clinical evaluations should be prioritized in future research to establish definitive guidelines for their safe and effective use.

Future advancements in 3D printing technology and material science could enhance resin formulations, post-curing protocols, finishing protocols, and multi-material printing, making 3D printed restorations a durable, cost-effective, and customizable alternative to traditional subtractive manufacturing in dentistry.

Translating laboratory findings into clinical practice presents challenges due to the complex oral environment, which differs from controlled in vitro conditions. Factors such as variable occlusal forces, saliva composition, thermal cycling, and patient habits, including bruxism, can accelerate material degradation. Additionally, differences in adhesive interface integrity, clinician technique, and post-processing protocols affect long-term performance. Since in vitro testing cannot fully replicate these variables, long-term clinical trials are essential to validate the durability and reliability of 3D printed materials in real-world applications.

## 5. Conclusions

The mechanical properties of 3D printed resins designed for permanent restorations remain lower than those of their subtractively manufactured counterparts. Moreover, even though these new 3D printed resins appear to be suitable for permanent single-tooth restorations, in the long term, the degradation processes that inevitably occur might significantly increase their chances of failure. Therefore, more laboratory and clinical studies are necessary to understand their clinical applications better. Additionally, standards for testing are advisable and cannot be postponed.

## Figures and Tables

**Table 1:** Search conducted in Medline/PubMed database.

Search	Query
#1	“print” [All fields] OR “manufactur*” [All fields] OR “technology” [All fields] OR “printed-resin” [All fields] OR “SLA” [All fields] OR “DLP” [All fields] OR “printing, three dimensional” [MeSH Terms]
#2	“crown” [All fields] OR “restoration” [All fields] OR “inlay” [All fields] OR “onlay” [All fields] OR “overlay” [All fields] OR “veneer” [All fields]
#3	“flexural” [All fields] OR “fracture load” [All fields] OR “hardness” [All fields] OR “elastic modulus” [All fields] OR “mechanic*” [All fields] OR “stress” [All fields] OR “compressive strength” [All fields] OR “Fracture resistance” [All fields] OR “fracture toughness” [All fields] OR “fatigue” [All fields] OR “Toughness” [All fields] OR “chip*” [All fields] OR “wear” [All fields] OR “mechanical properties” [All fields] OR “Mechanical processes” [All fields] OR “peak stress” [All fields] OR “Elastic strength” [All fields] OR “Microhardness” [All fields] OR “Brittleness” [All fields] OR “Flexibility” [All fields] OR “mechanical phenomena” [MeSH Terms] OR “stress, mechanical” [MeSH Terms] OR “Mechanical Tests” [MeSH Terms] OR “Flexural Strength” [MeSH Terms] OR “elasticity” [MeSH Terms] OR “elastic modulus” [MeSH Terms] OR “compressive strength” [MeSH Terms] OR “hardness” [MeSH Terms] OR “Hardness Tests” [MeSH Terms] OR “Dental Restoration Wear” [MeSH Terms]
#4	“composite” [All fields] OR “resin based” [All fields] OR “resin-based” [All fields] OR “resin” [All fields]
#5	“3D” [All Fields] OR “three dimensional” [All Fields] OR “addit*” [All Fields] OR “stereolithography” [All Fields] OR “stereolithography” [MeSH Terms]
#6	“interim” [All Fields] OR “provisional” [All Fields] OR “temporary” [All Fields]
#7	#1 AND #2 AND #3 AND #4 AND #5 AND NOT #6

**Table 2:** Risk of bias assessment according to QUIN.

Author, Year	Clearly Stated Aims/Objectives	Detailed Explanation of Sample Size Calculation	Detailed Explanation of Sampling Technique	Details of the Comparison Group	Detailed Explanation of Methodology	Operator Details	Randomization	Method of Measurement of Outcome	Outcome Assessor Details	Blinding	Statistical Analysis	Presentation of Results	Total	Score
Cakmak 2023 [17]	2	0	2	2	2	2	2	2	2	0	2	2	20	Low
Corbani 2020 [18]	2	0	1	0	2	2	0	2	0	0	2	2	13	Medium
Donmez 2022 [19]	2	2	2	1	2	1	1	2	1	0	2	2	18	Low
Grzbieluch 2021 [14]	2	1	1	2	2	1	2	2	0	0	2	2	17	Low
Guntekin 2024 [20]	2	2	2	1	1	1	1	2	0	0	2	2	16	Medium
Guven 2023 [21]	2	2	2	1	2	2	1	2	1	0	2	2	19	Low
Karaoglanoglu 2023 [22]	2	2	2	2	2	0	2	2	0	0	2	2	18	Low
Park 2024 [23]	2	2	2	2	2	0	0	2	0	0	2	2	16	Medium
Prause 2023 [24]	2	2	1	2	2	1	0	2	0	0	2	2	16	Medium
Suksuphan 2023 [25]	2	1	1	1	2	1	0	2	0	0	2	2	14	Medium
Türksayar 2023 [26]	2	2	1	2	2	0	1	2	0	0	2	2	16	Medium
Yilmaz, 2024 [27]	2	2	2	2	2	0	0	2	0	0	2	2	16	Medium
Zimmermann 2019 [28]	2	2	2	2	2	0	0	2	0	0	2	2	16	Medium

**Table 3:** Data extraction form.

Author, Year	Permanent 3D Printed Resin-Based Material Tested	Milled Resin-Based Material Tested	Printing Method	Printing Layer Orientation	Printing Layer Thickness (mm)	Specimen Shape	Specimen Thickness (mm)	Residual Resin Cleaning Method	Post-Printing Polimerization Method	Aging Procedures	Fatigue Simulation	Mechanical Properties Tested
Zimmermann, 2019 [28]	Els-3d Harz (Saremco Dental Ag)	Lava Ultimate (3m Espe); Cerasmart (GC Corporation); Brilliant Crios (Coltène)	DLP	N.r.	50	Crown	0.5; 1.0; 1.5	Ultrasonic bath in isopropanol 98% for 2 × 3 min	4000 lighting exposures using the Otoflash g171 device under a nitrogen oxide gas atmosphere.	N.r.	1.2 million cycles, 1.7 hz, invariable occlusal load 49 n ± 0.7 n, thermal cycling 5–55 degrees, dwell time 120 s, 12,000 cycles, water change time 10 s. Load was applied to the central fissure by the cusp of a natural molar.	Fracture resistance
Corbani, 2020 [18]	Irix Max (DWS)	Brilliant Crios (Coltène)	SLA	N.r.	50	Crown	0.5; 1.0; 1.5	95% ethanol for 1 min	Ultraviolet curing unit (Dcure; DWS) for 6 min	N.r.	5000 cycles in distilled water at 5 °C and 55 °C, with 30 s of dwell time and 5 s of transfer time. axial loading force of 50 n, frequency of 1.6 hz in the center of the occlusal surfaces with a stainless steel ball of 3-mm diameter for 1,200,000 cycles.	Fracture resistance
Grzebieluch, 2021 [14]	Varseosmile Crown Plus (Bego)	Grandio Blocs (Voco); Brilliant Crios (Coltène)	LCD/MSLA	Varseosmile group A: samples were printed vertically to the platform, without supports. Varseosmile group B: samples were rotated in the x and y axes by 45 degrees, with medium sized supports.	50	Bar	1.5	Ethanol (according to the user manual)	Final curing was performed in form cure (Formlabs), 2 times by 45 min (samples were rotated after the first exposure), with the temperature set on 0.	N.r.	N.r.	Flexural strength; Flexural modulus; Microhardness
Donmez M.B. 2022 [19]	Crowntec (Saremco Dental Ag)	Brilliant Crios (Coltène); Cerasmart 270 (GC Corporation)	DLP	Z compensation of 0 μm; XY compensation of 0 μm.	50	Crown	1.5–2.5	The external surfaces of the crowns were cleaned with an alcohol-soaked (96%) cloth; the internal surfaces were cleaned with a brush soaked in an alcohol solution.	4000 lighting exposures by using a xenon lamp-curing device (Otoflash g171; NK Optik) under nitrogen oxide gas atmosphere.	N.r.	N.r.	Fracture resistance
Karaoğlanoğlu S, 2023 [22]	Crowntec (Saremco Dental Ag); Permanent Crown (Formlabs)	Cerasmart 270 (GC Corporation); Grandio Blocs (Voco)	DLP; SLA	N.r.	50; 50	N.r.	N.r.	99% isopropyl alcohol; in an automatic washing machine (Form wash, Formlabs) using 99% isopropyl alcohol for 3 min.	Labolight duo (GC Corporation) for 6 min; Formcure (Formlabs) for 20 min at 60 °C	Specimens were immersed in beverages (tea, coffee, and water) for 30 days.	N.r.	Microhardness
Suksuphan P, 2023 [25]	Varseosmile Crown Plus (Bego)	Cerasmart 270 (GC Corporation)	DLP	N.r.	50	Crown	0.8; 1; 1.5	Ultrasonic baths	Post-cured according to the manufacturer’s recommendations.	N.r.	N.r.	Fracture resistance
Türksayar A.A.D. 2023 [26]	Crowntec (Saremco Dental Ag); Varseosmile Crown Plus (Bego)	Brilliant Crios (Coltène)	DLP	N.r.	50	Crown	N.r.	96% alcohol-soaked cloth; ultrasonic bath containing 96% ethanol solution for a total of 5 min (3 min of precleaning in reusable ethanol and 2 min of cleaning in fresh ethanol).	Otoflash g171 (NK Optik) under nitrogen oxide gas atmosphere (2000 × 2 lighting exposures); Otoflash g171 (NK Optik) under nitrogen oxide gas atmosphere (1500 × 2 lighting exposures).	N.r.	Distilled water under a 50-n load with 1.2 million cycles and 0.7-mm lateral sliding (5 °C to 55 °C, dwell time 30 s) by using a mastication simulator (chewing simulator CS-4.10; SD Mechatronik).	Fracture resistance; Wear behavior
Prause E. 2023 [24]	Varseosmile Crown Plus (Bego)	Grandio Blocks (Voco)	DLP	Vertically on the build platform at a distance of 2 mm above the platform.	50	Disc	1.5	Ultrasonically cleaned in 99% isopropanol, first for 3 min of rough cleaning, followed by 2 min of final cleaning.	Otoflash (Bego) for 2 × 1500 flashes	N.r.	N.r.	Flexural strength
Cakmak, 2023 [17]	Varseosmile Crown Plus (Bego); Crowntec (Saremco Dental Ag)	Brilliant Crios (Coltène)	DLP	N.r.	50	Disc	N.r.	Alcohol-soaked (96%) cloth; ultrasonically cleaned in ethanol for 5 min (3 min of precleaning in reusable ethanol and an additional 2 min in fresh ethanol).	4000 (ct, 2 × 2000) or 3000 (vs, 2 × 1500) light exposures with Otoflash g171 (NK Optik) under a nitrogen oxide gas atmosphere.	N.r.	25,000 cycles of artificial brushing (50,000 strokes, each cycle considered as a linear back-and-forth brushing action at a frequency of 1.5 hz) with an automatic brushing machine (bürstmaschine linear lr1); 10,000 thermal cycles (SD Mechatronik thermocycler; SD Mechatronik GMBH) at 5 °C–55 °C in a coffee solution with a dwell time of 30 s and a transfer time of 10 s.	Microhardness
Güven, 2023 [21]	Crowntec (Saremco Dental Ag)	Brilliant Crios (Coltène)	DLP	N.r.	50	Crown	1	N.r.	N.r.	N.r.	1.7 hz frequency for 1.2 million cycles under 50 n load with a lateral movement of 1 mm and a vertical and lateral sliding speed of 60 mm/s to simulate 5 years of functional loading. The load was applied with 3 mm stainless steel sphere antagonists, which contacted the central fossae of the crowns; simultaneously thermocycled for 6000 cycles between 5–55 °C with 30 s holding and 15 s transfer time in distilled water.	Wear behavior
Yilmaz 2024 [27]	Tera Harz Tc-80dp (Graphy); C and B Permanent (ODS)	Brilliant Crios (Coltène)	DLP	Long axis parallel to the platform, with the thinner side (1.5 mm) on the bottom.	50	Bar	1.5	Ultrasonic bath of 96% ethanol (ethanol absolut, Grogg chemie) for 45 s, and further cleansed with an ethanol-soaked (96% ethanol) cloth to remove any unpolymerized resin on the surface. To ensure that no alcohol residue was left on the specimens, they were then completely dried with an air syringe and allowed to dry thoroughly for at least 10 min.	2000 flash exposures, repeated twice on the top and bottom surfaces of each specimen with a xenon lamp-curing device (Otoflash g171, NK-Optik) under a nitrogen oxide gas atmosphere.	10,000 thermal cycles (thermocycler, sd mechatronik) from 5 °C to 55 °C with 10 s of transfer time (30-s dwell time) in distilled water.	N.r.	Flexural strength; Microhardness
Guntekin 2024 [20]	Permanent Crown Resin (FormLabs)	Cerasmart (GC Corporation); Grandio Blocks (Voco)	SLA	N.r.	N.r.	Square	2	Ultrasonic cleaner (Sonorex RK 102 Transistor, Bandelin) for 2 × 3 min with isopropyl alcohol (98%).	Light cured with an illumination exposure of 4000 lux under an atmosphere consisting of nitrogen oxide gas.	N.r.	Thermomechanical aging in a specially designed 8-chamber chewing device (Multifunctional chewing simulator, Analitik Medikal, Gaziantep, Turkey). Four hundred thousand cycles were completed under a vertical occlusal force of 49 ± 0.7 N with a thermal cycle of 1.7 Hz 5–55 °C and with a dwell time of 120 s, mimicking 2 years of aging.	Wear
Park 2024 [23]	Tera Harz Tc-80dp (Graphy); Permanent Crown Resin (FormLabs)	Mazic Duro (Vericom)	DLP; SLA	Occlusal plane parallel to the platform.	N.r.	Crown	0.5; 1.0; 1.5	95% isopropyl alcohol for 5 min in an ultrasonic cleaner (Shinhan 200H 3 L, Shinhan-sonic, Incheon, Republic of Korea) and then washed with a light-emitting diode (wavelength 405 nm and light intensity of 400 mW/cm).	Post-cured for 30 min in a polymerization device (Cure-M 102H, Sona Global Co., Ltd., Seoul, Republic of Korea).	N.r.	N.r.	Fracture resistance

N.r.: Not Reported.

**Table 4:** Materials’ compositions.

Product	Matrix	Filler	Manufacturer
Els-3d Harz	Not Available	Not Available	Saremco Dental Ag
Irix Max	Not Available	Inorganic fillers 42 wt. %	DWS
Varseosmile Crown Plus	Esterification products of 4,4′-isopropylidiphenol, ethoxylated and 2-methylprop-2enoic acid, methyl benzoylformate, diphenyl (2,4,6-trimethylbenzoyl)	Silanized dental glass, phosphine oxide, 30–50 wt. % inorganic fillers (particle size 0.7 μm)	Bego
Crowntec	Esterification products of 4,4’isopropylidiphenol, ethoxylated and 2-methylprop-2enoic acid, initiators	Inorganic silica fillers, 30–50 wt. % (particle size 0.7 μm)	Saremco Dental Ag
Permanent Crown	4,4′-isopropylidiphenol, ethoxylated and 2-methylprop-2enoic acid, methyl benzoylformate, diphenyl (2,4,6-trimethyl benzoyl)	Inorganic fillers, 30–50 wt. %(particle size 0.7 μm)	Formlabs
Tera Harz Tc-80dp	Urethane acrylate oligomer, bisphenol A ethoxylated dimethacrylate, 2-HEMA, diphenyl (2,4,6-trimethylbenzoyl)	Phosphine oxide and additives	Graphy
C and B Permanent	Diurethane dimethacrylate, 2-propenoic acid, 2-methyl-, (1-methylethylidene) bis (4, 1-phenyleneoxy [1-methyl-2, 1-ethanediyll) ester, 2-HEMA, diphenyl (2,4,6-trimethylbenzoyl)	phosphine oxide and additives	ODS

HEMA = hydroxyethyl-methacrylate.

**Table 5:** Fracture resistance (thickness 1, 1.5 mm).

Author, Year	Printing Method	Dye Material	Fatigue Simulation	Cross-Head Speed	3D printed Specimens’ Mean Fracture Load (N; 1 mm Thickness)	Milled Specimens’ Mean Fracture Load (N; 1 mm Thickness)	3D Printed Specimens’ Mean Fracture Load (N; 1.5 mm Thickness)	Milled Specimens’ Mean Fracture Load (N; 1.5 mm Thickness)
Zimmermann, 2019 [28]	DLP	SLA-printed resin	Yes	1 mm/min	els-3D: 1055.1 ± 133.8	Lava Ultimate: 831.4 ± 251.4; Cerasmart: 1170.2 ± 279.7; Brilliant Crios: 1255.7 ± 233.9;	els-3D: 1478.7 ± 168.2	Lava Ultimate: 1516.2 ± 282.9; Cerasmart: 1251.1 ± 430.9; Brilliant Crios: 1580.4 ± 521.0;
Corbani, 2020 [18]	SLA	SLA-printed resin	Yes	0.5 mm/min	Iris Max: 1945.9 ± 65.3	Brilliant Crios: 932.1 ± 41.2	Iris Max: 2383.5 ± 188.5	Brilliant Crios: 1284.7 ± 77.6
Donmez, 2022 [19]	DLP	Metal	N.r.	1 mm/min	/	/	Crowntec: 1413.9 ± 140.5	Brilliant Crios: 1333.2 ± 144.7; Cerasmart: 1274.3 ± 135.8;
Suksuphan, 2023 [25]	DLP	SLA-printed resin	N.r.	1 mm/min	Varseosmile Crown Plus: 1629.4 ± 118.5	Cerasmart: >2000	Varseosmile Crown Plus: 1747.2 ± 108.7	Cerasmart: >2000
Park, 2024 [23]	DLP, SLA	Titanium alloy 3D printing material	N.r.	1 mm/min	Tera Harz TC-80DP: 2677.3 ± 398.2; Permanent Crown: 1638.0 ± 148.1	Mazic Duro: 2326.3 ± 225.0	Tera Harz TC-80DP: 2176.8 ± 362.0; Permanent Crown: 1307.5 ± 102.0	Mazic Duro: 3218.8 ± 228.7

**Table 6:** Microhardness.

Author, Year	Printing Method	Type of Test	Specimen Polished	Printing Layer Orientation	3D Printed Specimens’ Mean Microhardness Values	Milled Specimens’ Mean Microhardness Values
Grzebieluch, 2021 [14]	LCD/LMSAD	Vickers	Composite rubbers HiLusterPlus^®^ Polishing System (Kerr Corp., Orange, CA, USA)	Varseosmile group A: samples were printed vertically to the platform, without supports. Varseosmile group B: samples were rotated in the x and y axes by 45 degrees, with medium size supports.	Varseosmile Crown Plus A: 25.8 ± 0.7; Varseosmile Crown Plus B: 28.16 ± 1.42	Grandio Blocks: 140.43 ± 5.47; Brilliant Crios: 75.40 ± 2.18
Karaoğlanoğlu, 2023 [22]	DLP/SLA	Vickers	A two-step diamond polishing kit (Clearfil Twist Dia, Kuraray, Japan)	N.r.	Crowntec: 30.0 ± 1.3; Permanent Crown: 37.4 ± 1.3	Grandio Blocks: 203.9± 3.6; Cerasmart: 103.8 ± 2.7
Cakmak, 2023 [17]	DLP	Vickers	Vareosmile Crown Plus and Crowntec: A slurry of coarse pumice in water (Pumice fine; Benco Dental) was used to conventionally polish one surface of all specimens for 90 s at 1500 rpm. Fine polishing was performed by using a polishing paste (Fabulustre; Grobet USA) for an additional 90 s	N.r.	Crowntec: 30.59 ± 2.95; Varseosmile Crown Plus: 34.57 ± 1.23	Brilliant Crios: 82.2 ± 7.08
Yilmaz, 2024 [27]	DLP	Vickers	A slurry of coarse pumice in water (Pumice fine; Benco Dental) was used to conventionally polish one surface of all specimens for 90 s at 1500 rpm. Fine polishing was performed by using a polishing paste (Fabulustre; Grobet USA) for an additional 90 s.	Long axis parallel to the platform, with the thinner side (1.5 mm) on the bottom.	Tera Harz Tc-80dp: 19.45 ± 1.68; C and B Permanent: 23.08 ± 4.7	Brilliant Crios: 85.39 ± 2.77

**Table 7:** Flexural strength (thickness 1.5 mm).

Author, Year	Type of Test	Printing Method	Specimen Thickness (mm)	Cross-Head Speed	Printing Layer Orientation	3D Printed Specimens’ Mean Flexural Strength Values (Mpa)	Milled Specimens’ Mean Flexural Strength Values (Mpa)
Grzebieluch, 2021 [14]	Three-point bending test	LCD/LMSAD	1.5	1 mm/min	Varseosmile group A: samples were printed vertically to the platform, without supports. Varseosmile group B: samples were rotated in the x and y axes by 45 degrees, with medium sized supports.	Varseosmile Crown Plus A: 119.85 ± 17.95; Varseosmile Crown Plus B: 143.39 ± 12.88	Grandio Blocks: 186.02 ± 10.49; Brilliant Crios: 170.29 ± 9.41
Prause, 2023 [24]	Biaxial bending test	DLP	1.5	N.r.	Vertically on the build platform at a distance of 2 mm above the platform.	Varseosmile Crown Plus: 83.5 ± 18.5	Grandio Blocks: 237.3 ± 31.6
Yilmaz, 2024 [27]	Three-point bending test	DLP	1.5	1 mm/min	Long axis parallel to the platform, with the thinner side (1.5 mm) on the bottom.	Tera Harz Tc-80dp: 107.55 ± 15.69; C and B Permanent: 90.82 ± 4.33	Brilliant Crios: 211.35 ± 16.98

**Table 8:** Sensitivity analysis.

Metric	Full Dataset Mean	Low Risk Only Mean	Difference
Fracture Resistance (N)	1641.54	1630.00	11.54
Flexural Strength (MPa)	126.38	126.60	−0.22
Microhardness (VHN)	30.54	31.00	−0.46
Wear Behavior (µm)	50.15	51.40	−1.25

**Table 9:** Correlation analysis between filler content (%) and key mechanical properties.

Metric	Filler Content (%)	Fracture Resistance (N)	Flexural Strength (Mpa)
Filler Content (%)	1.00	0.88	0.67
Fracture Resistance (N)	0.88	1.00	0.08
Flexural Strength (Mpa)	0.67	0.08	1.00

## Data Availability

No new data were created or analyzed in this study. Data sharing is not applicable to this article.

## Supplementary Materials

The following supporting information can be downloaded at: https://www.mdpi.com/article/10.3390/ma18050985/s1. Support Information: Preferred Reporting Items for Systematic Reviews and Meta-Analyses Extension for Scoping Reviews (PRISMA-ScR) Checklist.
